# The proliferating cell hypothesis: a metabolic framework for *Plasmodium* growth and development^☆^

**DOI:** 10.1016/j.pt.2014.02.001

**Published:** 2014-04

**Authors:** J. Enrique Salcedo-Sora, Eva Caamano-Gutierrez, Stephen A. Ward, Giancarlo A. Biagini

**Affiliations:** 1 Liverpool School of Tropical Medicine, Pembroke Place, Liverpool, L3 5QA, UK; 2 Warwick Systems Biology Centre, Senate House, University of Warwick, Coventry, CV4 7AL, UK

**Keywords:** Warburg effect, glycolysis, malaria, epigenetics, gametocytes, dormancy

## Abstract

We hypothesise that intraerythrocytic malaria parasite metabolism is not merely fulfilling the need for ATP generation, but is evolved to support rapid proliferation, similar to that seen in other rapidly proliferating cells such as cancer cells. Deregulated glycolytic activity coupled with impaired mitochondrial metabolism is a metabolic strategy to generate glycolytic intermediates essential for rapid biomass generation for schizogony. Further, we discuss the possibility that *Plasmodium* metabolism is not only a functional consequence of the ‘hard-wired’ genome and argue that metabolism may also have a causal role in triggering the cascade of events that leads to developmental stage transitions. This hypothesis offers a framework to rationalise the observations of aerobic glycolysis, atypical mitochondrial metabolism, and metabolic switching in nonproliferating stages.

## Highlights

• The hypothesis offers a framework to explain the atypical features of parasite metabolism.
• Aerobic glycolysis is hypothesised to meet the biosynthetic demands of rapid proliferation.
• Differentiation may be epigenetically regulated in response to nutrient-linked metabolism.

## Aerobic glycolysis drives proliferation in single-minded eukaryotes

Rapidly proliferating eukaryotes have perfected metabolic modes that efficiently convert glucose and specific amino acids into biomass (see Glossary) and energy at the required pace. The past decade has brought a change in the accepted paradigm on accelerated cell multiplication. Streamlined metabolic networks and the capacity to support anabolic reactions in a rapidly responsive manner via aerobic fermentative glycolysis and glutaminolysis, instead of pursuing thorough oxidation of the glycolytic carbons via cellular respiration, seems to be a precondition for rather than a consequence of effective proliferative signalling [1]. The corollary of this paradigm points to respiration in nonproliferating cells as the prevalent metabolic mode to generate the energy needed to perform their roles as differentiated cells.

## Current concept of the Warburg effect

Although originally ascribed to anaerobic metabolism, the preference for fermentative glycolysis even under aerobic conditions was accepted long ago as a feature in cancer cells and is known as the Warburg effect [2]. Similarly, *Saccharomyces cerevisiae* favour fermentation over respiration when glucose is available even under oxygen abundance (Crabtree effect) [3]. In its original form, the Warburg effect also stated that the oxidation of glucose in mitochondria was ablated. However, more recent evidence points to functional mitochondrial oxidative phosphorylation in some cancer cell lines [3,4]. Under this modern version of the Warburg effect, rapidly proliferating, noncancerous cells have also been found to undergo aerobic glycolysis/fermentation [5–7].

**Figure 1:**
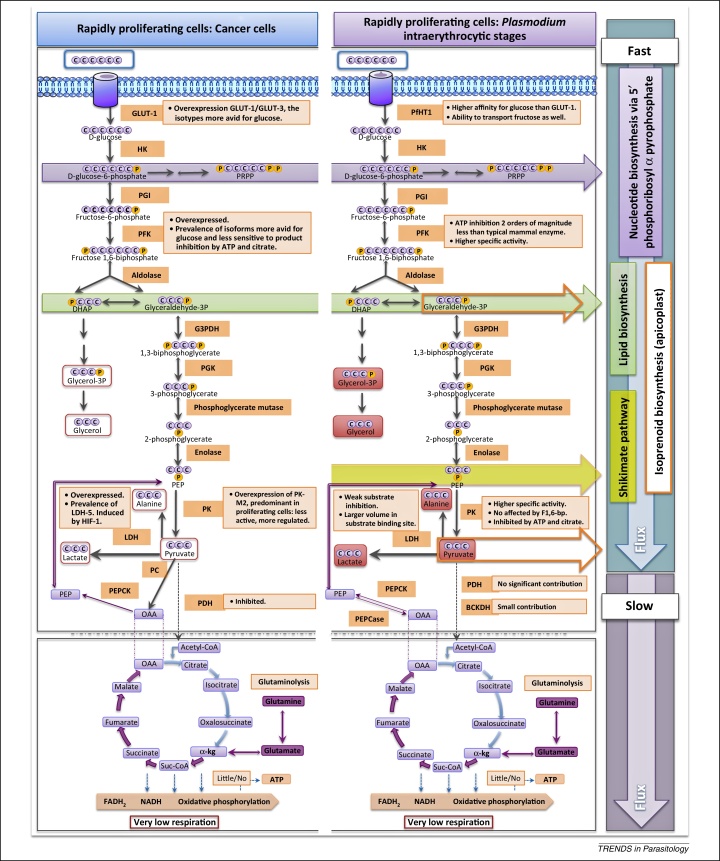
Proliferating cell hypothesis: similarities between cancer cells and *Plasmodium falciparum*. Principle end products of glucose consumption (lactate, alanine, pyruvate, glycerol-3-phosphate, and glycerol, shown in red boxes) are similar in both cancer cells [3] and asexual intraerythrocytic malaria parasites [12]. A high glycolytic flux maintains rate-limiting glycolytic intermediates to support nucleotide (via glucose-6-phosphate to 5-phosphoribosyl-α-pyrophosphate) and lipid biosynthesis (via dihydroxyacetone phosphate to glycerol-3-phosphate). Metabolic modifications (Boxes 1 and 2) allow aerobic glycolysis/fermentation to proceed rapidly whilst keeping tricarboxylic acid (TCA) flux low. Anapleorotic glutaminolysis follows past part of the TCA cycle through the five-carbon α-ketoglutarate [15]. Subsequent conversion of oxaloacetate to phosphoenolpyruvate (PEP) by phosphoenolpyruvate carboxykinase (PEPCK, EC 4.1.1.49) allows for further synthesis of biosynthetic intermediates (e.g., via shikimate pathway [16] and isoprenoid biosynthesis [17]). Abbreviations: GLUT-1, glucose transporter 1; PfHT1, *Plasmodium falciparum* hexose transporter 1; HK, hexokinase (EC 2.7.1.1); PGI, phosphoglucose isomerase (EC 5.3.1.9); PFK, phosphofructokinase (EC 2.7.1.11); G3PDH, glyceraldehyde 3 phosphate dehydrogenase (EC 1.2.1.12); PGK, phosphoglycerate kinase (EC 2.7.2.3); PK, pyruvate kinase (EC 2.7.1.40); LDH, lactate dehydrogenase (EC 1.1.1.27); PEPCase, phosphoenolpyruvate carboxylase (EC 4.1.1.31); PC, pyruvate carboxylase (EC 6.4.1.1); PDH, pyruvate dehydrogenase (EC 1.2.4.1); BCKDH, branched chain ketoacid dehydrogenase (EC 1.2.4.4); Suc-CoA, succinyl-CoA.

The advantage provided to rapidly proliferating cells by increased glycolysis is attributed to the capacity of glucose to support biomass generation by redirection of glycolytic intermediates into anabolic reactions while at the same time sustaining a predominant (over 90%) fermentation flux to lactate [3,5,7,8] (Figure 1, Boxes 1 and 2). The latter is necessary for the regeneration of NAD^+^, an essential cofactor of glycolysis itself, but more importantly and less intuitively, to allow the cells to gauge their metabolic status. Thus, only when high levels of fermentative glycolysis are possible does the cell enter high rates of proliferation assisted by the anabolic capacity of glycolysis.

## Aerobic glycolysis during the *in vitro* cell cycle of *Plasmodium falciparum*

The intraerythrocytic cycle of human falciparum malaria takes the parasites through successive rounds of mitosis every 48 h. Following erythrocyte invasion by a merozoite, but sometimes following multiple invasions, the parasite develops into a ring-shaped form in the first 24 h, and by approximately 30 h, the parasite very rapidly expands to occupy most of the space available within the erythrocyte plasma membrane, resulting in a major increase in biomass. From approximately 40 h, the vastly enlarged nucleus goes through several asynchronous and multiple segmentations that *in vitro* produce a number (small double figures) of next-generation merozoites [9]. Cytokinesis occurs near the end of the cycle before the new daughter cells (merozoites) emerge as free-living forms for seconds to minutes in the search for a new erythrocyte [9]. A fraction, usually less than 1% but dependent on the prevailing environment, of the newly generated intraerythrocytic parasites are programmed to differentiate as gametocytes, the sexual nondividing forms that in the natural environment continue the malaria cycle in the mosquito vector [10].

Malaria parasites committed to proliferation in the intraerythrocytic cycle are fermentative organisms [11–13] (Figure 1, Boxes 1 and 2) with an anabolic central carbon metabolism that can feed all major biomass generating pathways [14]. When directed to differentiation into gametocytes, however, these nonproliferative cells seem to follow the respiration of glucose in a manner more in line with the biology of eukaryotes in stationary phase via the canonical glucose-driven, mitochondrial tricarboxylic acid (TCA) cycle. Current evidence appears to substantiate this dichotomy of fermentation when in proliferation mode versus respiration when committed to sexual differentiation [15].

In proliferating asexual parasites, glutaminolysis feeds part of the TCA cycle through the five-carbon α-ketoglutarate. The four-carbon malate and oxaloacetate are transported to the cytoplasm. Here phosphoenolpyruvate (PEP) can be synthesised from oxaloacetate by the activity of phosphoenolpyruvate carboxykinase (PEPCK) for onward biosynthetic reactions (e.g., shikimate pathway [16] and isoprenoid biosynthesis [17]) (Figure 1). In nonproliferating gametocytes whereby a more canonical glucose TCA cycle is present, less glucose is catabolised by fermentation to lactate, and minimal glutamine is catabolised by glutaminolysis [15].

The paradigm of the rapidly proliferating eukaryote can then be applied to profile the dividing intraerythrocytic *P. falciparum* as an organism that in the presence of abundant glucose and glutamine, such as the levels available in human plasma, generates the required biomass by aerobic glycolysis/fermentation and glutaminolysis (Figure 1, Boxes 1–3). The rest of the macromolecular biomass is salvaged from the purine precursors, amino acids, and lipids or fatty acids of the human host. Under these conditions, a low flux glycolytic TCA cycle and a modified electron transport chain provides a further selective advantage (Boxes 1 and 2).

## Are there metabolic regulatory switches controlling life cycle commitment in *Plasmodium*?

The established dogma states that *Plasmodium* metabolism is simply a functional consequence of the ‘hard-wired’ genome-wide, just-in-time regulation of expression [18,19]. However, there is increasing evidence in biology to support the notion that metabolism, in response to the environment/diet, can be causal, promoting the switch of cellular phenotypes. Examples in nature range from post-translational modifications (PTMs) of histones by constituents of royal jelly (fatty acids) causing larvae to become queens instead of worker bees [20], to PTMs of histones in the Agouti viable yellow mouse model, whereby different maternal methyl-donor supplementation (e.g., with folic acid, vitamin B12, or betaine) results in different offspring ranging from obese hyperinsulinaemic yellow to leaner nonhyperinsulinaemic pseudoagouti phenotypes [21].

The malaria parasite controls vital virulence processes such as host cell invasion and cytoadherence, at least in part, by epigenetic mechanisms [22]. With this in mind, and given that *in vitro* and *in vivo* nutrient/stress conditions have been linked with life cycle commitment in *Plasmodium* [23–25], it is not inconceivable that parasite metabolism may promote changes in phenotype via one or more of the many metabolites that are known to influence epigenetic gene regulation in other cell types.

In cancer cells and yeast, for example, nutrient availability and metabolic status, including the yeast metabolic cycle (YMC) fluctuating from oxidative phosphorylation and fermentation, is coupled to the control of gene expression via key metabolites such as NAD^+^, acetyl Co-A, FAD, and folates [26–28].

The influence of metabolism on parasite epigenetics is certainly an exciting area for future research, and some evidence, although circumstantial, exists to link nutrient levels to parasite development. Environmental stress has been consistently correlated with enhanced gametocyte production both *in vitro* and *in vivo*. The methodology applied to enrich *in vitro* cultures of *P. falciparum* with sexual forms has the common denominator of nutrient deprivation: low haematocrit, haemoglobin depletion, lysed erythrocytes, and recycling of spent media, among others [23,29]. Antimalarials that act as antimetabolites such as antifolates have long been known to increase gametocyte production *in vivo* [24]. *In vivo* transcriptional profiles of *P. falciparum* blood stages show that a proportion of the parasite population appears to be in states similar to what is known as either a starvation response or environmental stress in yeast [25]. Therefore, natural variability of substrate levels in the human host, perhaps not surprisingly, seems to be a selective force for life cycle commitment pathways in field populations of *Plasmodium*. Unfortunately, cellular metabolism of malaria parasites under variable nutrient availability has been poorly investigated, a situation not helped by the routine use of highly enriched media normally used for the *in vitro* culture of *P. falciparum* [30].

The decision of a parasite to commit to a sexual lineage is believed to take place in the first 20 h (the ‘ring’ stage) of the preceding erythrocytic cycle [29]. Interestingly, the early ring stages of *P. falciparum* have less compact histone cores (nucleosomes) than in later stages [9], and usually this ‘open’ conformation is reflective of, and conducive to, transcriptional regulation. As in other organisms and cell types it is therefore possible that in *Plasmodium* there exists a metabolic component that controls, via an epigenetic mechanism, the commitment to replicate or to differentiate.

A further, metabolically controlled, decision-making option open to the parasite in the early hours of intracellular parasite life is the possibility of reversible cell cycle arrest. As part of their parasitic lifestyle, *P. falciparum* become dependent on the extracellular supply of isoleucine due to an absence of this amino acid in human haemoglobin. Media that lacks isoleucine induce reversible cell cycle arrest with parasites not progressing beyond the first half, the ring stage, of their asexual intraerythrocytic life cycle unless the missing nutrient is provided [31]. In malaria, the phenomenon of reversible cell cycle arrest is poorly understood. Nonetheless, there is a new interest in studying malaria dormancy in the intraerythrocytic stages of the parasite life cycle due to the potential role of reversible cell cycle arrest in the slow clearance and/or ring stage survival (RSA_0–3h_) phenotypes seen in clinical failures with artemisinins [32–35].

## Concluding remarks

Glucose and glutamine contribute to malaria parasite biomass for the biosynthesis of nucleotides and lipids via aerobic glycolysis/fermentation and glutaminolysis. Together with salvaged amino acids, fatty acids, and purines, these are the main biochemical resources used to assemble the macromolecular structure of the plasmodial cell. However, there are two further options available: (i) differentiation into a sexual lineage as gametocytes and (ii) cell cycle arrest. The first half of the intraerythrocytic cycle of *P. falciparum*, particularly within the initial 10 h, seems to be the stage at which quorum sensing and decision making is most relevant. As seen with other organisms and cell types, we have discussed the possibility that this occurs via nutrient/metabolite-dependent epigenetic mechanisms. Deconvolution of these regulatory processes offers a new and exciting chapter in our understanding of *Plasmodium* biology (Box 4).

## Box 1 Metabolic rewiring for rapid parasite proliferation: glycolysis

Glucose entry into the parasite occurs via the hexose transporter PfHT1, which has a *K*_m_ of ∼0.5 mM. Compared with the ∼5 mM blood glucose concentration, this allows for a constant rate of transport [36]. Commitment to glycolysis is then controlled via the highly regulated phosphofructokinase (PFK) that is allosterically inhibited by high levels of ATP. In cancer cells, PFK is overexpressed, and the predominant isoforms of this enzyme possess allosteric alterations that reduce the degree of product inhibition by ATP and citrate whilst being more highly activated by lower concentrations of fructose 2,6-bisphosphate (F26bP) [37–39]. In *P. falciparum* PFK, deregulation is also observed with the enzyme being insensitive to PEP, citrate, and F26bP and only exhibiting allosteric behaviour for ATP and ADP, although at elevated concentrations (>1.0 mM for ATP and >0.1 mM for ADP) [40]. The final irreversible step in glycolysis involves pyruvate kinase (PK), generating pyruvate and ATP. This is a critical step in the control of biosynthetic intermediates for proliferation, and the enzyme is activated by fructose 1,6-bisphosphate and inhibited by both ATP and alanine. There are two isoforms in mammals, M1 and M2. M1 is found in adult tissue and is largely unregulated by fructose 1,6-bisphosphate and ATP, whereas the M2 isoform predominates in proliferating cells including cancer cells and is less active and more tightly regulated [41]. Tight regulation of PK is hypothesised to aid the control of flow of carbons between biosynthesis and lactate production in proliferating cells. Indeed, cancer cells engineered to express the M1 isoform produce more lactate [42,43]. *P. falciparum* PK is not activated by fructose 1,6-bisphosphate but is markedly inhibited by both ATP and citrate, akin to M2 mammalian isoforms [44].

## Box 2 Metabolic rewiring for rapid parasite proliferation: TCA and respiration

Pyruvate is a critical metabolic mode for entry into fermentation or the TCA cycle. For fermentation, pyruvate must remain in the cytosol, whereas for entry into the TCA cycle, pyruvate must enter mitochondria in order to be converted to acetyl-CoA. In proliferating cells, where described aerobic glycolysis is required for the generation of biosynthetic intermediates, cells have evolved mechanisms which either: (i) restrict the transport of pyruvate into mitochondria [45]; (ii) inhibit pyruvate dehydrogenase (PDH) activity [46]; or (iii) increase the activity of lactate dehydrogenase [47]. There is no information concerning pyruvate transport into the mitochondrion of *P. falciparum*; however, the parasite does contain PDH, but this is localised to the apicoplast and does not appear to contribute to the acetyl-CoA pool [48]. A mitochondrially localised complex, termed branch chain ketoacid dehydrogenase (BCKDH), with PDH-like activity, has been hypothesised to contribute acetyl-CoA to the TCA, this notwithstanding; however, labelling experiments indicate that the rate of acetyl-CoA production is significantly slower compared with the labelling of glycolytic intermediates [48]. Lactate production in *P. falciparum* is extensive and in line with other key parasite glycolytic enzymes, and lactate dehydrogenase activity is deregulated, exhibiting only weak inhibition by pyruvate or by the pyruvate/NAD^+^ complex [49,50].

Defects of electron transport chain components also appear to be a feature in cancer cells. These include defects at the level of succinate dehydrogenase (SDH), inhibition of ATP synthase, and downregulation of complex I (NADH:dehydrogenase), III (*bc*_1_ complex), and IV (cytochrome *c* oxidase) [51–53]. *P. falciparum* also possess atypical mitochondrial function, whereby mitochondria have low O_2_ consumption and are not actively synthesizing ATP (respiratory state 4) [54]. Several adaptive features, including the absence of a transmembrane proton pumping complex I, enable proton-uncoupled oxidation of NADH, thereby reducing proton ‘back-pressure’ in the absence of extensive ATP synthesis. This in turn reduces mitochondrial superoxide generation and potential DNA damage and, importantly for glycolysis, still allows deregulated oxidation of cytosolic NADH [54]. The reported essentiality of complex V (ATP synthase [55]) is consistent with the need of a small H^+^ leak in order to maintain transmembrane H^+^ pumping by complexes III and IV [54].

## Box 3 Growing fast while fermenting furiously: crunching the numbers

Aerobic glycolysis is able to provide the required biosynthetic intermediates for building biomass, explaining why *Plasmodium* and other proliferating organisms and cell types adopt increased glucose metabolism during rapid growth and multiplication. By way of illustration, the capacity of *Plasmodium* to synthesise some of the required DNA precursors relates to the *de novo* synthesis of the pyrimidine deoxythymidine triphosphate (dTTP). The *de novo* synthesis of dTTP requires folate 5,10-methylene tetrahydrofolate (5,10-myTHF). In its final polyglutamated form, with five glutamic residues as found in an average eukaryote, 5,10-myTHF is a structure of 40 carbons and 11 nitrogens that requires two NADPHs and ten ATPs for its biosynthesis from GTP, d-erythrose-4-phosphate (E4P) and PEP (shikimate pathway [16]). Only two molecules of glucose are needed to contribute seven carbons and the two NADPHs (pentose phosphate pathway). The rest of the carbon count originates from five glutamates and a serine or glycine. The nitrogen sources are GTP (six nitrogens) and glutamate (five nitrogens) from glutaminolysis. Thus, the synthesis of 5,10-myTHF from glucose and GTP can be abbreviated as: 2 glucose (carbon) + 1 GTP + 5 glutamate + 5 glucose (ATP) + 1 serine/glycine → 1 (5,10-myTHF) + 5 ADP + glycine/(CO_2_ + NH_3_). Malaria parasites salvage precursors for the synthesis of purines such as GTP from the host as well as amino acids from plasma and the digestion of the haemoglobin of the host. Then, for every 100 molecules of glucose, if 90% are used to sustain a high fermentative glycolytic flux, where the needed ATP originates in abundance, ten molecules of glucose can be used to build up to five molecules of 5,10-myTHF. Human plasma contains a strictly regulated level of glucose to ∼5 mM, the equivalent of 3 × 10^15^ molecules of glucose per microlitre. That would be enough to build up to 7.5 × 10^14^ molecules of 5,10-myTHF per microlitre, the equivalent to 625 to 62.5 times what is needed to support an expected intracellular folate concentration in *P. falciparum* of approximately 2–20 μM.

## Box 4 Outstanding questions

• As described here, our hypothesis is that metabolism in the malaria parasite is highly evolved to promote rapid proliferation, in a similar manner to that seen in other rapidly proliferating cells, for example, cancer cells, activated lymphocytes, and yeast.
• The major ‘step change’ for future research questions will be to determine if metabolism can be causal. This will necessitate a deeper understanding of the metabolic nodes and checkpoints used by the parasite during growth and in response to its environment in its various hosts.

## Glossary

Aerobic glycolysis: predominant fermentation of glucose even under oxygen pressures considered to be aerobic. Fractions of glycolytic intermediates that are not fermented are redirected and are seemingly sufficient to sustain biosynthetic pathways such as the pentose phosphate pathway, shikimate pathway, and lipid biosynthesis.
Agouti viable yellow mouse model: heterozygous mice for the Agouti yellow allele have yellow coats and have a predisposition towards obesity. Mice that are homozygous for the Agouti yellow allele have the lethal gene. Mice that are homozygous for the non-agouti allele and non-agouti yellow allele have non-agouti coat colour such as black. In this model, coat colour variation is correlated to epigenetic marks established early in development, and is used extensively to investigate the impacts of nutritional and environmental influences on the (foetal) epigenome.
Anabolic reactions: relating to the synthesis of complex molecules in living organisms.
Anaerobic metabolism: relating to metabolism that occurs in the absence of free oxygen, often via substrate level phosphorylation and/or alternative terminal acceptors.
Anaplerosis: the process of replenishment of depleted metabolic cycle or pathway intermediates. Most commonly referring to the TCA cycle, this concept is also used to describe glycolysis and glutaminolysis generated substrates for macromolecular biosynthesis or anabolism.
Biomass: the total quantity or weight of organisms in a given area or volume. The measurement of biomass production is important when studying metabolic reactions that are required for growth.
Dormancy and reversible cell cycle arrest: cell quiescence, hibernation, dormancy, or reversible cell cycle arrest are denominations of a common and important physiological response in free-living microorganisms to control cell size and growth that grants protection against environmental insults including poor nutrient and micronutrient levels.
Fermentative glycolysis: breaking of glucose into different possible final products from the reduction of pyruvate as common intermediate. The better-known products are lactate in mammalian cells and ethanol in yeast. Replenishment of NAD^+^ is a crucial consequence of fermentation.
Glutaminolysis: alternative source of biomass and electrons due to the relative abundance of glutamine in human plasma. After deamination of this amino acid, glutamate feeds part of the TCA cycle. Intermediates such as malate and oxaloacetate can transit to the cytoplasm from mitochondria and be decarboxylated to replenish glycolytic pyruvate with the production of NADPH.
One-carbon mitochondrial metabolism: exchange of one carbon molecules at different levels of oxidation between folate intermediates catalysed by enzyme complexes loosely attached to the inner mitochondrial membrane. The glycine cleavage system (GCV), serine hydroxymethyltransferase (SHMT), and 5,10-methenyltetrahydrofolate dehydrogenase multienzyme complex (MTHFD) are their main components.
